# Short amplicon reverse transcription‐polymerase chain reaction detects aberrant splicing in genes with low expression in blood missed by ribonucleic acid sequencing analysis for clinical diagnosis

**DOI:** 10.1002/humu.24378

**Published:** 2022-04-27

**Authors:** Htoo A. Wai, Matthew Constable, Cosima Drewes, Ian C. Davies, Eliska Svobodova, Esther Dempsey, Anand Saggar, Tessa Homfray, Sahar Mansour, Sofia Douzgou, Kate Barr, Catherine Mercer, David Hunt, Andrew G. L. Douglas, Diana Baralle

**Affiliations:** ^1^ Human Development and Health, Faculty of Medicine, Southampton General Hospital University of Southampton Southampton UK; ^2^ Department of Experimental Biology, Faculty of Science Masaryk University Brno Czech Republic; ^3^ Department of Clinical Genetics St George's University of London London UK; ^4^ Manchester Centre for Genomic Medicine, St Mary's Hospital, Manchester University NHS Foundation Trust Health Innovation Manchester Manchester UK; ^5^ Division of Evolution and Genomic Sciences, Faculty of Biology, Medicine and Health, School of Biological Sciences University of Manchester Manchester UK; ^6^ Bristol Regional Clinical Genetics Service St Michael's Hospital Bristol UK; ^7^ Wessex Clinical Genetics Service University Hospital Southampton NHS Foundation Trust Southampton UK

**Keywords:** aberrant splicing, blood RNA, RNA‐seq, RT‐PCR, VUS

## Abstract

Use of blood RNA sequencing (RNA‐seq) as a splicing analysis tool for clinical interpretation of variants of uncertain significance (VUSs) found via whole‐genome and exome sequencing can be difficult for genes that have low expression in the blood due to insufficient read count coverage aligned to specific genes of interest. Here, we present a short amplicon reverse transcription‐polymerase chain reaction(RT‐PCR) for the detection of genes with low blood expression. Short amplicon RT‐PCR, is designed to span three exons where an exon harboring a variant is flanked by one upstream and one downstream exon. We tested short amplicon RT‐PCRs for genes that have median transcripts per million (TPM) values less than one according to the genotype‐tissue expression database. Median TPM values of genes analyzed in this study are *SYN1 *= 0.8549, *COL1A1 *= 0.6275, *TCF4 *= 0.4009, *DSP* = .2894, *TTN *= 0.2851, *COL5A2 *= 0.1036, *TERT *= 0.04452, *NTRK2 *= 0.0344, *ABCA4 *= 0.00744, *PRPH *= 0, and *WT1 *= 0. All these genes show insufficient exon‐spanning read coverage in our RNA‐seq data to allow splicing analysis. We successfully detected all genes tested except *PRPH* and *WT1*. Aberrant splicing was detected in *SYN1, TCF4, NTRK2, TTN*, and *TERT* VUSs. Therefore, our results show short amplicon RT‐PCR is a useful alternative for the analysis of splicing events in genes with low TPM in blood RNA for clinical diagnostics.

## 1 INTRODUCTION

RNA splicing analysis is increasingly being used to aid rare disease diagnosis through the detection of splicing variants (Murdock et al., 2021; Rowlands et al., 2020; H. A. Wai et al., 2020). This can be achieved either via targeted reverse transcription‐polymerase chain reaction (RT‐PCR) when testing the effects of known variants of uncertain significance (VUSs) or increasingly also via transcriptome‐wide RNA sequencing (RNA‐seq) (H. A. Wai et al., 2020). The limits of sensitivity of transcriptome‐wide RNA‐seq are yet to be fully defined or standardized but they depend upon tissue‐specific gene expression values, levels of transcript degradation, library preparation methods, sequencing parameters, and subsequent bioinformatic processing such as read filtering and alignment.

Transcripts per million (TPM) values can be calculated from RNA‐seq data and can be used to provide an estimate of whether sufficient coverage is likely to be achieved for specific genes of interest (Wagner et al., 2012). Publicly available expression datasets such as the TPM values from the genotype‐tissue expression (GTEx) project are frequently referred to when trying to determine whether or not splicing can be assayed via whole blood RNA sampling (Lonsdale et al., 2013). However, significant experimental protocol differences between prior published data and ongoing current RNA analyses may lead to errors in predicting the detectability of abnormal splicing if such datasets are relied upon in isolation.

Conventional RT‐PCR is known to be useful for the analysis of splicing as an alternative cost‐effective technique to RNA‐seq (Macken et al., 2021; H. A. Wai et al., 2020). Here, we sought to examine whether genes with low TPM values on RNA‐seq can still be assayed for potentially abnormal splicing through the use of RT‐PCR. We confirmed that exonic and junction‐spanning read coverages are poor in genes with low TPM values. We then used RT‐PCR to test VUSs for their effect on splicing in low TPM genes which cannot be readily analyzed using RNA‐seq, designing short‐amplicon PCR assays to see if they could outperform blood RNA transcriptome analysis for such genes and produce clinically actionable results.

## 2 MATERIALS AND METHODS

### 2.1 Patient recruitment procedure and RNA extraction

A total of 13 samples with low TPM genes from patients with VUSs were identified through the Splicing and Disease Research Study at the University of Southampton, UK, ethics approved by the Health Research Authority (IRAS Project ID 49685, REC 11/SC/0269) and by the University of Southampton (ERGO ID23056). Informed consent for splicing studies was provided for all patients from whom samples were obtained. Patient blood samples were collected using PAXgene Blood RNA tubes and intracellular RNA extraction from whole blood was performed with the PAXgene Blood RNA Kit (PreAnalytix). All the patient RNA sample's quality and concentration were checked with bioanalyzer before performing RT‐PCR and RNA‐seq.

### 2.2 RNA‐seq and data analysis

Whole transcriptome sequencing with ribosomal RNA depletion and stranded library preparation was carried out via Novogene using NovoSeq 600 PE150. A minimum coverage depth of 70M 150 base pair paired‐end reads were produced for each sample. Distribution of sequencing quality, error rate distribution, A/T/G/C distribution, the composition of raw data, and data quality summary were mentioned in the supplementary data (Data S1). Data analysis was performed on the IRIDIS 4 high‐performance computer cluster, University of Southampton. FASTQ reads were aligned to the reference human genome version 38 (GRCh38) using the STAR alignment tool (2.5.2b version) (Dobin et al., 2013) with reading level filters: reads corresponding to a mapping quality of 255 and maximum mismatch of 6. These filtering parameters were used to match those employed by GTEx. Filtered aligned BAM files were visualized and sashimi plots were generated using the integrative genomics viewer (Ttir et al., 2013).

### 2.3 RT‐PCR and polymerase chain reaction

All the experiments were conducted in the laboratory of Human Development and Health, Southampton General Hospital, University of Southampton. Complementary DNA (cDNA) was synthesized using the High‐Capacity cDNA Reverse Transcription Kit (Thermo Fisher Scientific) using random hexamers. Primer pairs (Data S) were designed using the online Primer3web tool (primer3.ut.ee), selecting binding sites in exons adjacent to variants and standard desalted synthetic single‐stranded DNA oligo primers were ordered from Integrated DNA Technologies. Polymerase chain reaction (PCR) experiments were performed using the GoTaq G2 Polymerase PCR system (Promega) and GoTaq Hot Start Polymerase (Promega) according to the manufacturer's protocol. PCR cycles were set up at 95°C for 5 min followed by 35 and 40 repetitive cycles of 95°C for 30 s, 60°C or the annealing temperature of individual primer pairs for 30 s and 72°C for 1 min per kilobase pair before ending the cycle with 72°C for 10 min and cooling down at 4°C. RT‐PCR products were purified by GeneJET PCR Purification Kit (Thermo Fisher Scientific) and bidirectional Sanger sequencing was carried out by SourceBioscience. Where indicated, amplicons for further analysis were gel‐purified by GeneJET Gel Extraction Kit (Thermo Fisher Scientific) and sequenced at SourceBioscience for Sanger sequencing. All the PCR experiments were repeated at least twice for reproducibility and all expected positive amplicons were confirmed by Sanger sequencing.

## 3 RESULTS

### 3.1 RNA‐seq sensitivity is reduced for low TPM genes

**Figure 1:**
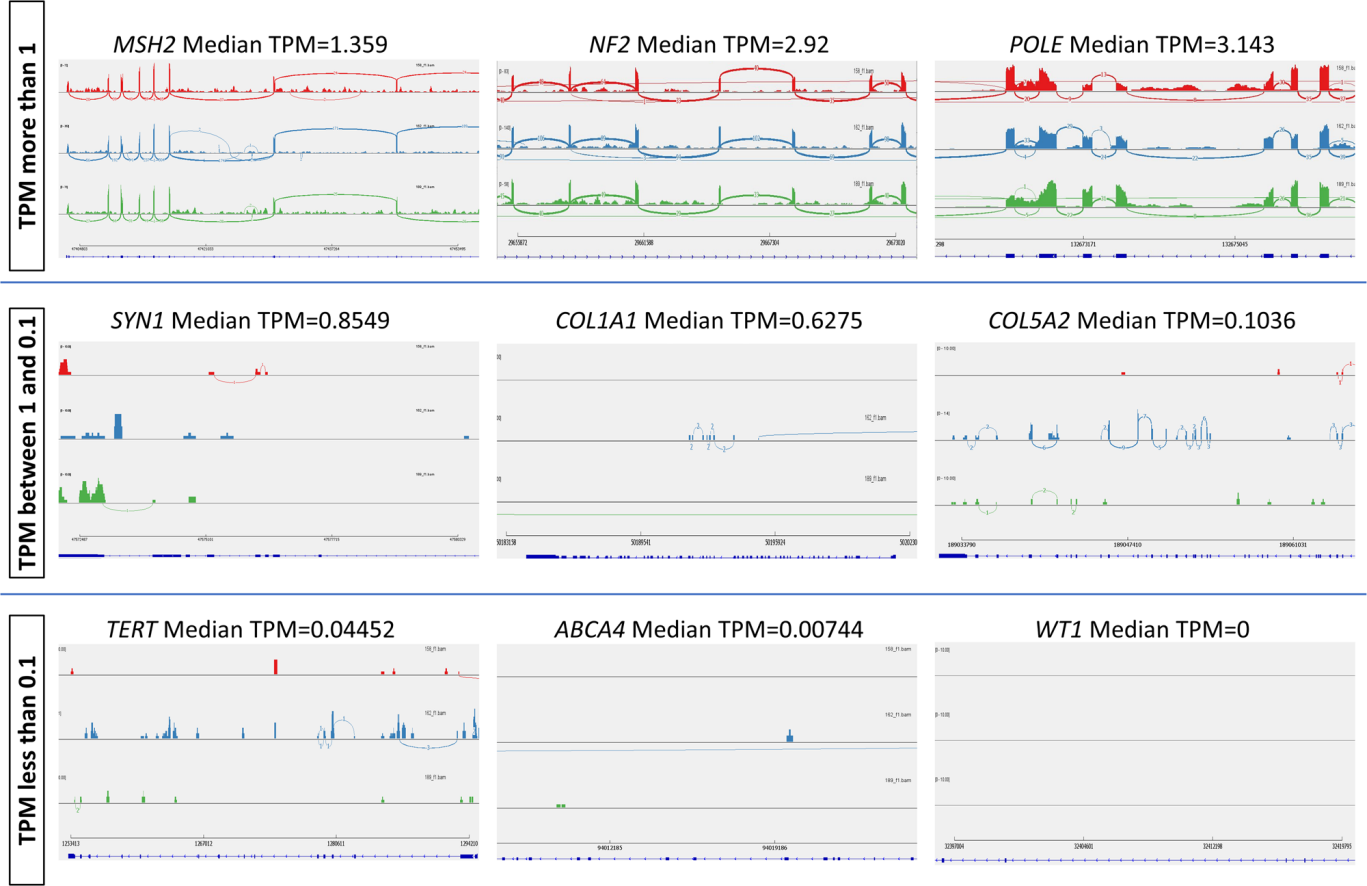
Sashimi plots showing junction‐spanning read comparison between genes with a median TPM value of more than 1 and genes with a median TPM value of less than 1 in data from three randomly selected blood RNA‐seq samples. Genes with a median TPM value of more than 1 have more exon spanning reads whereas genes with a median TPM value of less than 1 have less or no exon spanning reads. RNA‐seq, RNA sequencing; TPM, transcripts per million

TPM is a normalized RNA abundance measurement of high‐throughput RNA sequencing data. The values are calculated using the number of reads aligned to a gene of interest and they come from their respective genes or transcripts for every one million RNA molecules in the RNA‐seq sample (Wagner et al., 2012). It is a useful indicator of gene expression level and for predicting the detectability of splicing events using reads spanning exon–exon junctions. Exon‐spanning reads are crucial for splicing analysis using RNA‐seq and these are abundant in genes where the median TPM value is more than 1 (Figure 1). Aberrant splicing events can also be readily visualized in genes where the TPM value is 1 or more in RNA‐seq of blood RNA. However, exonic and exon‐spanning reads of genes with median TPM below 1 show lower or no read coverage (Figure 1). Therefore, aberrant splicing events can be missed due to insufficient read coverage where TPM values are lower than 1 and cannot be easily analyzed using the RNA‐seq.

### 3.2 Short amplicon RT‐PCR is more sensitive than standard RT‐PCR in detecting genes with low TPM in the blood

**Figure 2:**
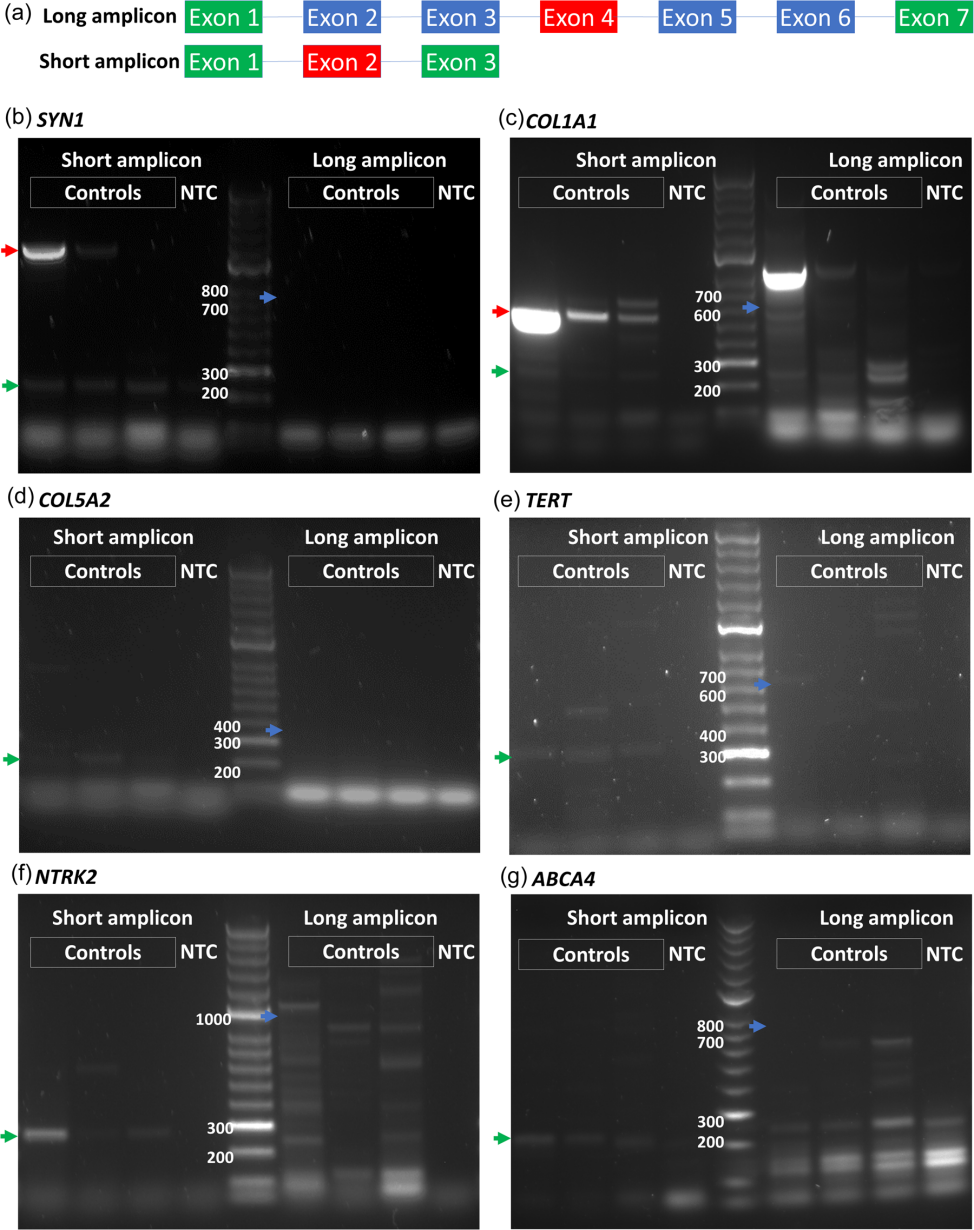
Short amplicon RT‐PCR has higher sensitivity than long amplicon RT‐PCR. (a) Long amplicon primers are designed to span at least seven exons whereas short amplicon primers are designed to span only three exons in RT‐PCR to detect low TPM genes. Red exons represent VUS harboring exons and green is the primer‐targeting exons. (b–g) Short amplicon and long amplicon PCRs are compared using three controls and one no template control (NTC) in *SYN1, COL1A1, COL5A2, TERT, NTRK2*, and *ABCA4*. Expected short and long amplicon sizes are indicated with green and blue arrows, respectively. Amplified genomic DNA fragments are indicated with red arrows. All PCR reactions were performed using GoTaq G2 DNA Polymerase (Promega) at 35 amplification cycles. RT‐PCR, reverse transcription‐polymerase chain reaction; TPM, transcripts per million; VUS, variants of uncertain significance

The primers we designed for conventional RT‐PCR for the detection of aberrant splicing are located so as to span at least seven exons, where the exon harboring the variant is at the center of the amplicon. In this way, aberrant splicing events such as one or two exon skipping affecting upstream or downstream exons with respect to the variant of interest can be detected by gel electrophoresis. This design of PCR for aberrant splicing detection is sensitive to genes that have high TPM values in the blood (Figure 2a). However, it fails to produce any amplicons for genes that have lower TPM values (less than 1) (Figure 2b–g). On the other hand, a short amplicon RT‐PCR design, in which primers span only three exons, is sensitive enough to amplify the targeted cDNA (Figure 2b–g) when performed at 35 cycles using GoTaq G2 DNA polymerase. As only three exons are spanned in short amplicon PCR, only single‐exon‐skipping aberrant splicing events can be detected and two exon‐skipping events may be missed.

Both short and long amplicons were further analyzed using different PCR conditions such as amplification cycles and polymerases. Two different amplification cycles, 35 and 40 cycles, and two different Taq DNA polymerases, GoTaq G2 and GoTaq Hot Start polymerases, were tested for the three genes with low TPM values. Only short amplicons were detected in all different PCR conditions whereas nonspecific amplicons or no amplicons were detected in long amplicon PCR reactions, confirming that short amplicon PCR has higher sensitivity than a long one (Figure S1).

### 3.3 Short amplicon RT‐PCR detects normal and aberrant transcripts of genes with low TPM values

**Figure 3:**
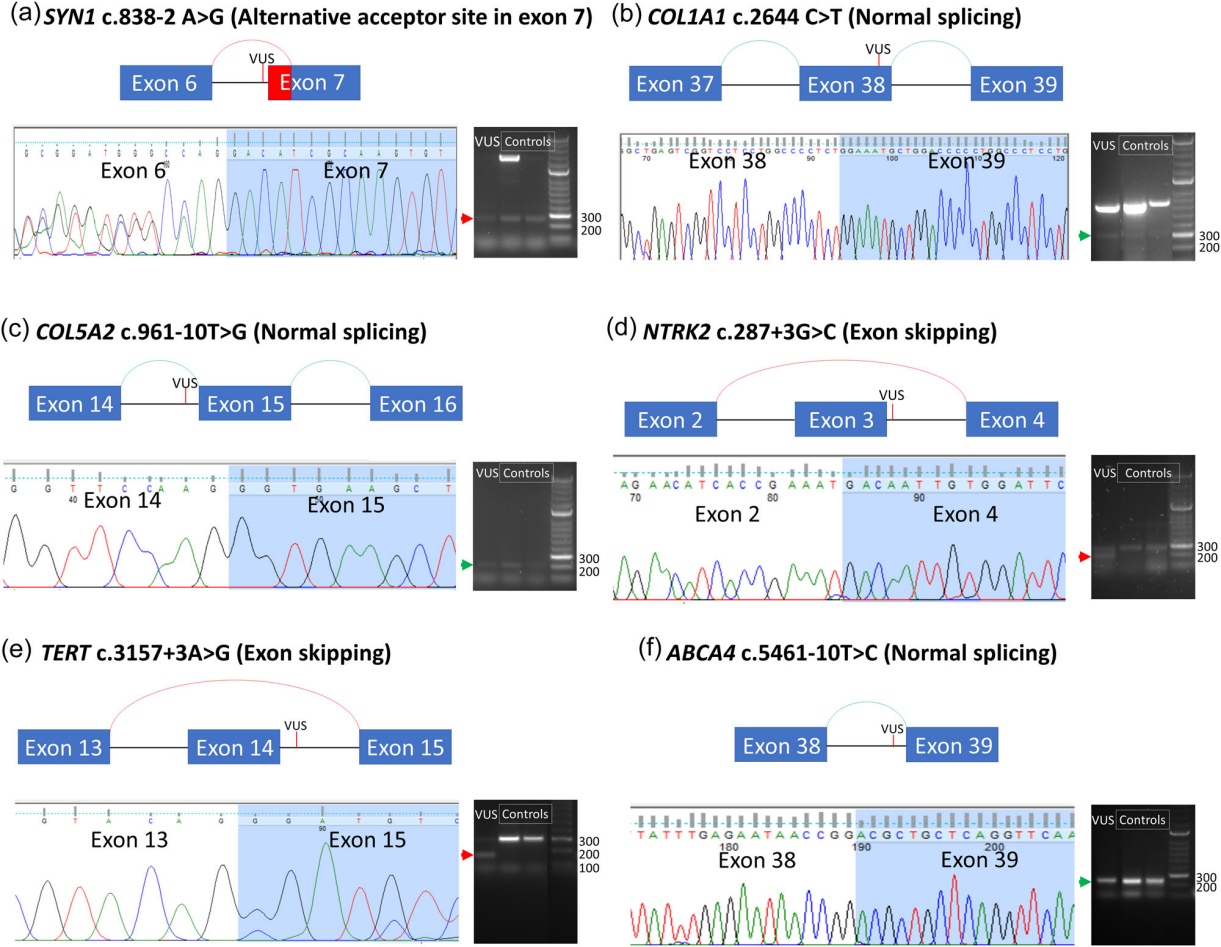
Gel electrophoresis and Sanger sequencing results of short amplicon RT‐PCR for low TPM genes. (a–c) Examples of three genes with TPM ranging from 1 to 0.1, *SYN1* (TPM = 0.8459), *COL1A1* (TPM = 0.6275), and *COL5A2* (TPM = 0.1036). (d,e) Examples of two genes with TPM ranging from 0.1 to 0.01, *TERT* (TPM = 0.04452) and *NTRK2* (TPM = 0.0334). (f) Example of a gene with TPM ranging from 0.01 to 0.001, *ABCA4* (TPM = 0.00744). Green arrows and arches indicate the normal splicing amplicons and events whereas the red arrow and arches highlight the aberrant splicing amplicons and events. Gels were spliced to form the images shown in the figure and the editors have reviewed the respective raw data. RT‐PCR, reverse transcription‐polymerase chain reaction;TPM, transcripts per million

Four different categories of low TPM genes were analyzed using short amplicon RT‐PCRs. These categories were based on the GTEx database median TPM values: 1 to 0.1 (*COL1A1, COL5A2*, *DSP, SYN1*, *TCF4*, *TTN*), 0.1 to 0.01 (*NTRK2*, *TERT)*, 0.01 to 0.001 (*ABCA4*), and 0 (*PRPH, WT1*). Amplicons were successfully detected in genes with median TPM ranges from 1 to 0.001 using short amplicon RT‐PCR (Figure 3). Aberrant splicing events were detected in *SYN1* c.838‐2 A>G (median TMP= 0.8549) (Figure 3a), *TCF4* c.550‐3C>G (median TPM = 0.4009), *NTRK2* c.287+3G>C (median TPM = 0.03444) (Figure 3d), *TERT* c.3295+5G>T (median TPM = 0.04452), *TERT* c.5137+3A>G (median TPM = 0.04452) (Figure 3e), *TTN* c.49346‐1G>A (median TPM = 0.2851), and *TTN* c.63793G>A (median TPM = 0.2851). In comparison, sashimi plots of RNA‐seq read coverage data across splice junctions for low TPM gene regions show poor or no exonic or exon‐spanning reads covering these areas. The results of all VUSs tested are summarized in Table 1. Amino acid changes caused by aberrant splicing are also shown in Table 1.

**Table 1:** VUS information of low TPM genes and their aberrant splicing results

Gene	cDNA	Protein	RefSeq ID	Chr	Coordinates (g38)	Strand	SNV position from splice junction (A = acceptor, D = Donor)	Splicing result	Splice abberration
*SYN1*	c.838‐2 A>G	p.(=)	NM_006950.3	X	47576641	Reverse	A‐2	Exonic A5SS	r.838_867del, p.(Val280_Gln289del)
*COL1A1*	c.2644C>T	p.(Arg882Ter)	NM_000088.3	17	50189702	Reverse	D‐24	Normal	
*TCF4*	c.550‐3C>G	p.(=)	NM_001083962.2	18	55279658	Reverse	A‐3	Exon skipping	r.550_655del, p.(Val184MetfsTer15)
*DSP*	c.5510A>G	p.(Asn1837Ser)	NM_004415.4	6	7582772	Forward	A+130	Normal	
*TTN*	c.49346‐1G>A	p.(=)	NM_001267550	2	178613938	Reverse	A‐1	Exon skipping	r.49346_49532del, p(Asp16449GlufsTer2)
*TTN*	c.63793G>A	p.(Asp21265Asn)	NM_001267550	2	178587516	Reverse	D‐1	Intron retention and exonic A5SS	r.63793_63794ins63793+1_63794‐1, p.(Asp21265SerfsTer14); r.63592_63793del, p.(Val21198ThrfsTer9)
*COL5A2*	c.961‐10T>G	p.(=)	NM_000393.5	2	189079117	Reverse	A‐10	Normal	
*TERT*	c.3295+5G>T	p.(=)	NM_198253.3	5	1254363	Reverse	D+5	Exon skipping	r.3158_3295del, p.(Gly1053AlafsTer35)
*TERT*	c.3157+3A>G	p.(=)	NM_198253.3	5	1255284	Reverse	D+3	Intronic A3SS and exon skipping	r.3157_3158ins3158‐159_3158‐1, p.(Gly1053AlafsTer45); r.3033_3157del, p.(Phe1012AspfsTer123)
*NTRK2*	c.287+3G>C	p.(=)	NM_006180.6	9	84702236	Forward	D+3	Exon skipping	r.213_287del, p.(Ile71_Leu96del)
*ABCA4*	c.5461‐10T>C	p.(=)	NM_000350.3	1	94011395	Reverse	A‐10	Normal	
*PRPH*	c.421G>T	p.(Met293Ile)	NM_006262.4	12	49295621	Forward	D‐125	No product	
*WT1*	c.871A>T	p.(Ser291Cys)	NM_024426.6	11	32427972	Reverse	D‐17	No product	

Abbreviations: cDNA, complementary DNA; Chr, chromosome; SNV, single‐nucleotide variant; TPM, transcripts per million; VUS, variant of uncertain significance.

## 4 DISCUSSION

RNA‐seq is being integrated into clinical diagnostic services as a tool for the identification of pathogenic sequence variants not identified by standard exome or genome data filtering (Douglas & Baralle, 2021). However, using RNA‐seq to detect aberrant splicing patterns in genes not well expressed in the tissue source can be difficult due to low read coverage of exons and splice junctions. In our RNA‐seq analysis, blood RNA was used to generate 70 million, 150 bp paired‐end reads per sample. However, 70 million read coverage is not enough to generate exonic and junction spanning reads for low TPM genes. A possible solution to overcome low read counts is to increase the total read counts generated by RNA‐seq. However, the cost of sequencing increases prohibitively for large‐scale testing in a healthcare system. In addition, the generation of more read counts per sample may still not guarantee successful splicing analysis in low TPM genes, due to individual differences in gene expression (Lonsdale et al., 2013). A solution would be to source the relevant cells or tissues that have high gene expression levels of the genes of interest. However, alternative tissue sources are often not feasible or pleasant for the patient, highlighting the importance of a reliable source of RNA for splicing analysis.

Conventional RT‐PCR‐based splicing analysis has been traditionally used for the assessment of VUSs and their effect on splicing (H. Wai et al., 2019; H. A. Wai et al., 2020). However, even this technique can fail in genes where there is low TPM. In contrast, we found that our redefined short amplicon RT‐PCR, spanning only three exons, is sensitive enough to detect low TPM genes, providing crucial clinically useful information. However, one drawback is that, unlike long amplicon PCR, short amplicon RT‐PCR may miss multiexon skipping events.

Although aberrant splicing is associated with disease phenotype, the phenotypic severity may depend on the type of aberrant splicing. For example, in‐frame aberrant splicing may have milder phenotypic effects than a frameshift or termination codon induced by aberrant splicing, depending on the relevant gene's pathogenetic mechanisms (Lord & Baralle, 2021). However, further studies are needed on a gene‐by‐gene basis to determine what usage level of alternative splicing is pathogenically significant.

RNA‐seq read coverage for low TPM genes becomes sparse (TPM value 1 to 0.01) and completely disappears for genes in which TPM values are lower than 0.01. We found that short amplicon RT‐PCR can detect genes beyond this threshold with TPM ranging from 1 to 0.001. Minigene assays are an alternative method for analyzing variants not detected by either RNA‐seq or RT‐PCR but these are time‐consuming to set up and are not easily translated into a high‐throughput diagnostic service (Pagani & Baralle, 2009). In contrast, this short RT‐PCR method can be applied in a more high‐throughput manner for aberrant splicing detection in the majority of genes of interest.

In conclusion, our findings highlight the limits of diagnostic RNA‐seq for low TPM genes. Redefined short amplicon RT‐PCR is a method that is cheaper, simpler, and quicker. Our findings show that short RT‐PCR amplicons compensate for the shortfalls of RNA‐seq in assessing splicing in low TPM genes, down to 0.001 TPM but not where TPM is zero, as a useful adjunct to large‐scale transcriptomics for genomic diagnostic services.

## AUTHOR CONTRIBUTIONS

Htoo A. Wai developed, designed, conducted the experiments, and wrote and revised the manuscript. Matthew Constable, Cosima Drewes, Ian C. Davies, and Eliska Svobodova contributed to the experiments. Esther Dempsey, Tessa Homfray, Anand Saggar, Sahar Mansour, Sofia Douzgou, Kate Barr, Stephanie Greville‐Heygate, and David Hunt provided the patient samples. Andrew G. L. Douglas and Diana Baralle supervised the study and edited, and revised the manuscript. Andrew G.L. Douglas is co senior author with Diana Baralle.

## CONFLICTS OF INTEREST

The authors declare no conflicts of interest.

## Supporting information

Supporting information.

## Data Availability

Variants of uncertain significance information were submitted to the ClinVar database (https://www.ncbi.nlm.nih.gov/clinvar/) with accession numbers from SCV002106369 to SCV002106379.
