# Supplementary material for: Short amplicon reverse transcription‐polymerase chain reaction detects aberrant splicing in genes with low expression in blood missed by ribonucleic acid sequencing analysis for clinical diagnosis
Source: Hum Mutat. 2022 Apr 27;43(7):963–70. doi: 10.1002/humu.24378 (PMC9325405; doi:10.1002/humu.24378)
Supplement: Supplementary file 1 — Supporting information. [file HUMU-43-963-s001.docx]

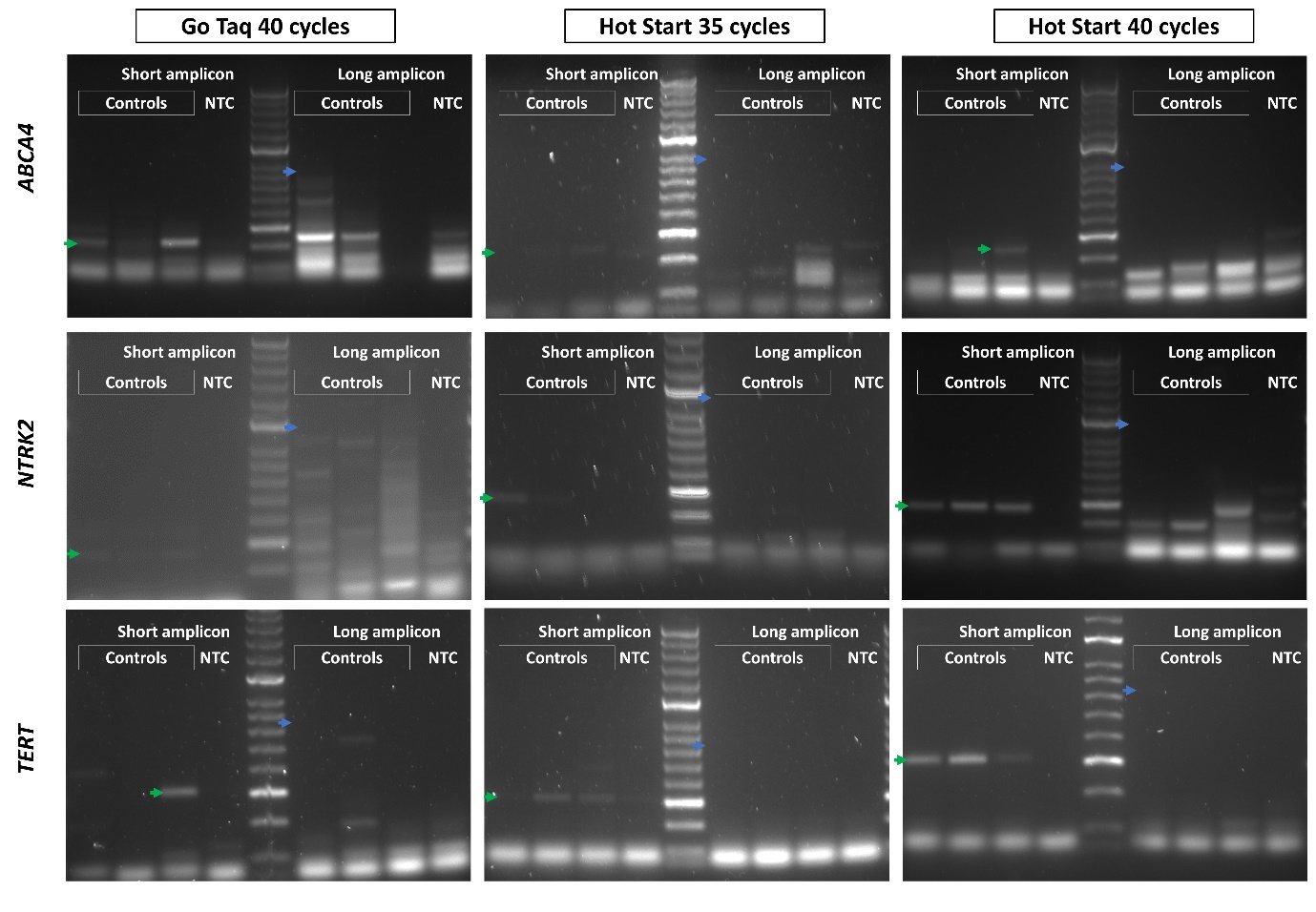


**Supporting Figure S1. Comparisons of short and long amplicons RT-PCR for *TERT, NRTK2* and *ABCA4* under different PCR conditions.** Reactions with GoTaq G2 polymerase for 40 cycles, Go Taq Hot Start polymerase with 35 cycles and 40 cycles were tested for the three genes with TPM values less than 1. Short amplicons were detected but long amplicons were absent in all three conditions.

**Supporting Data D1**

SOT189


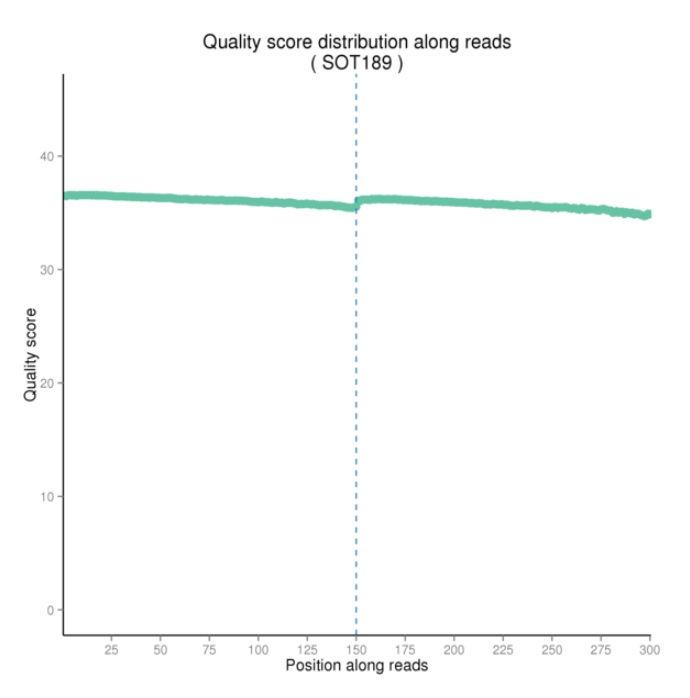

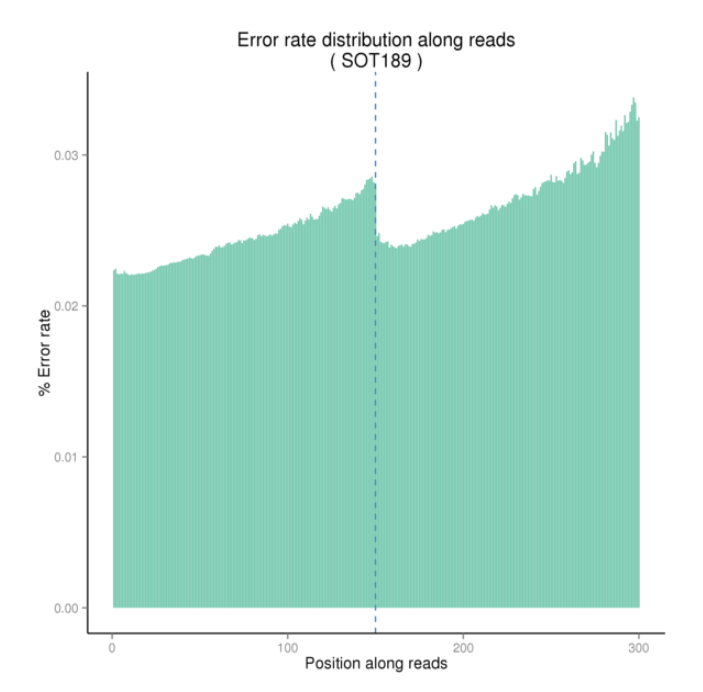


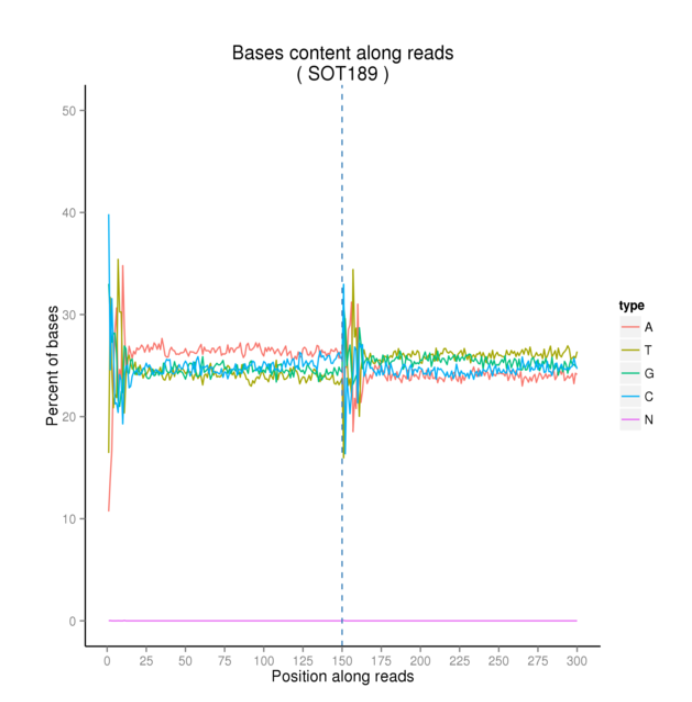

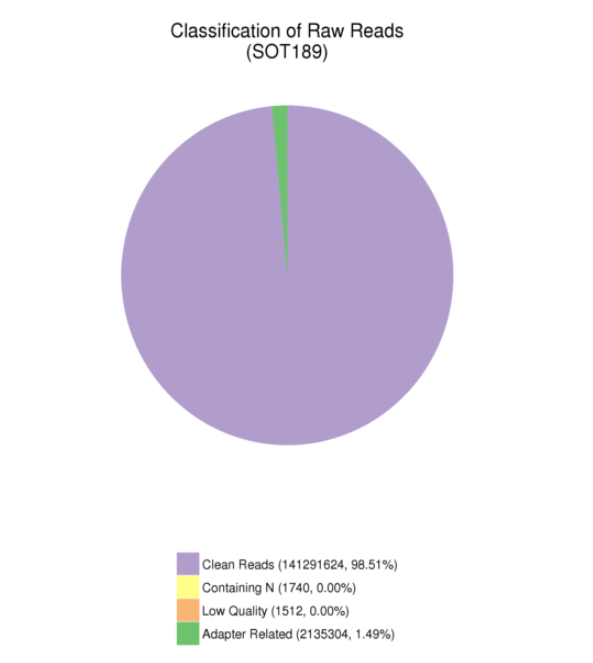


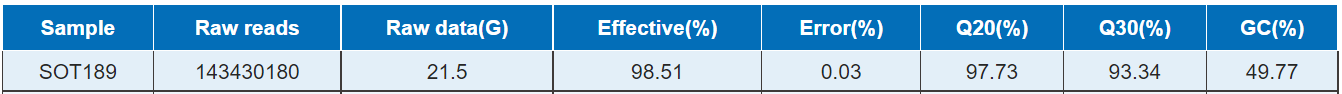


SOT158


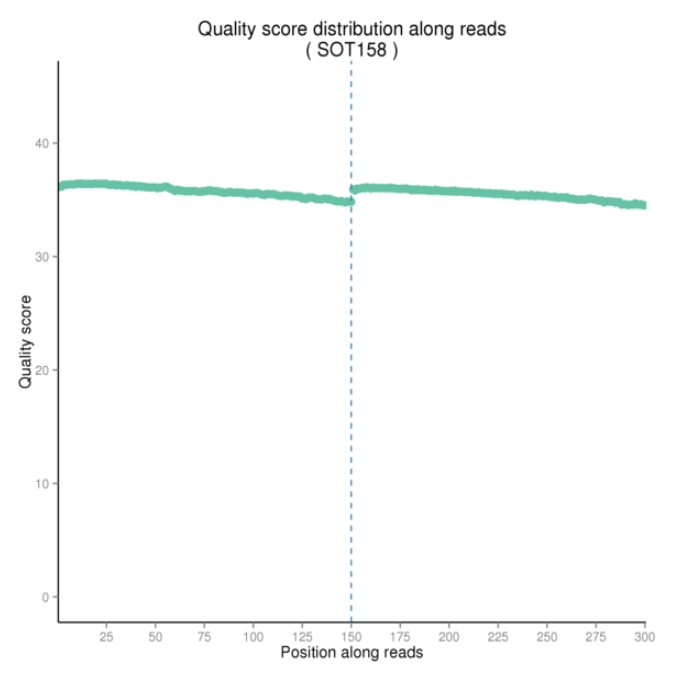

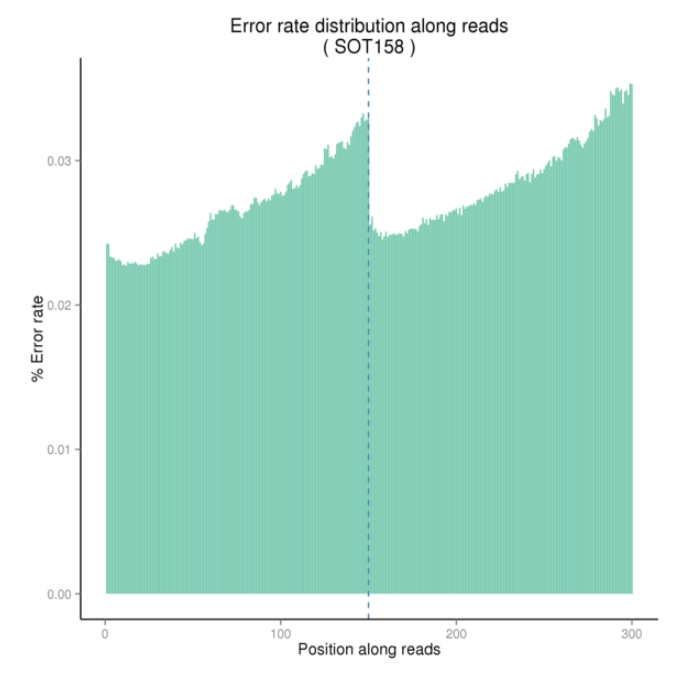

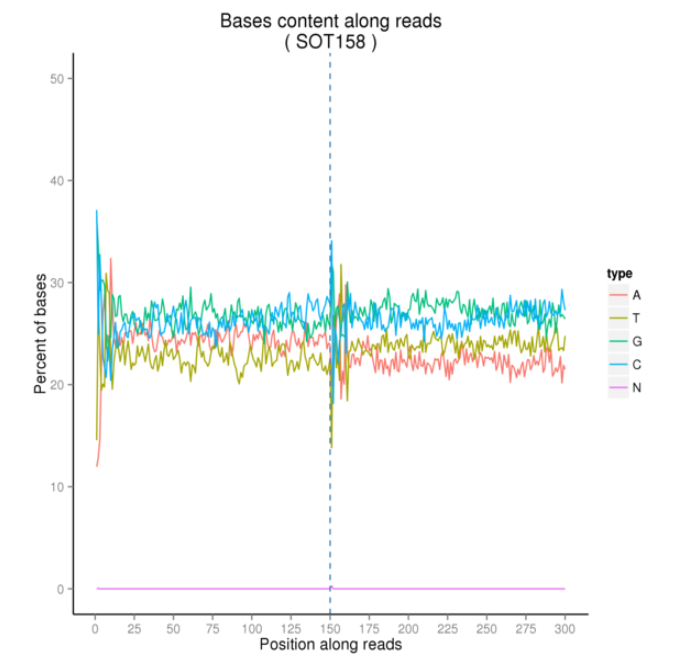

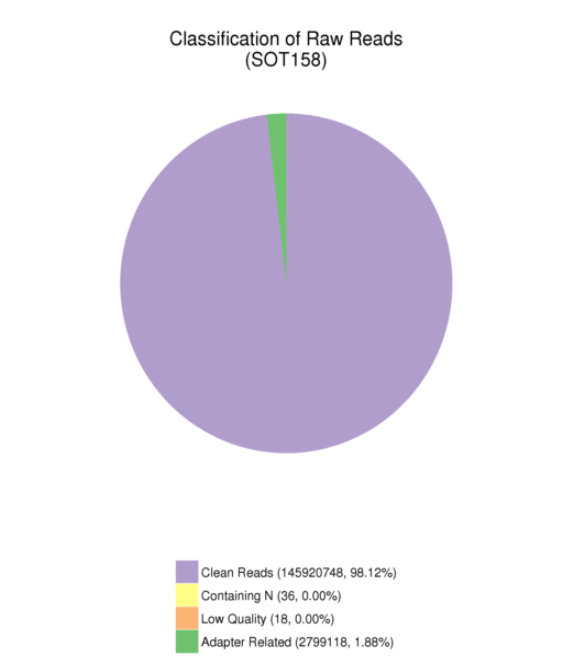


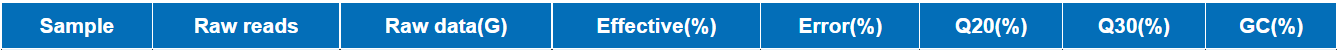

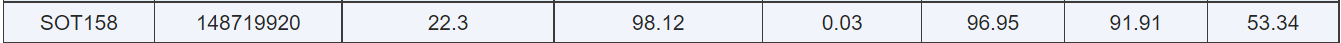


SOT168


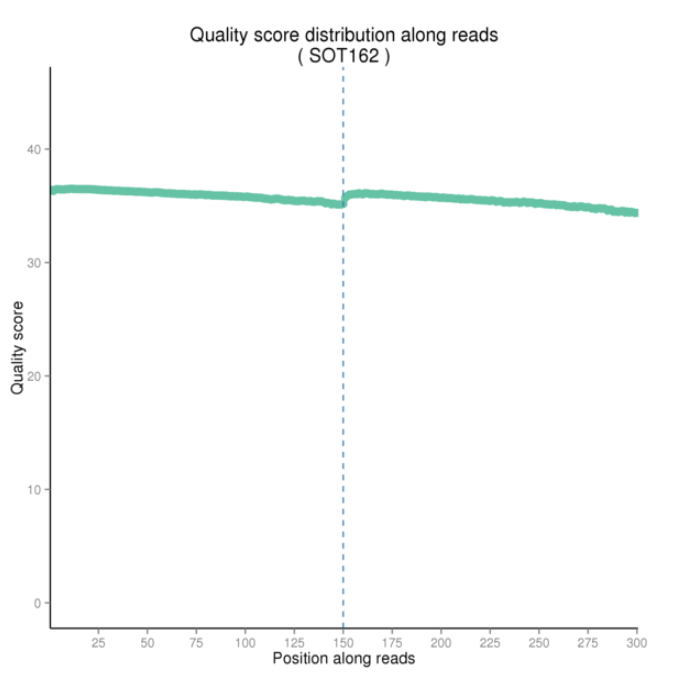

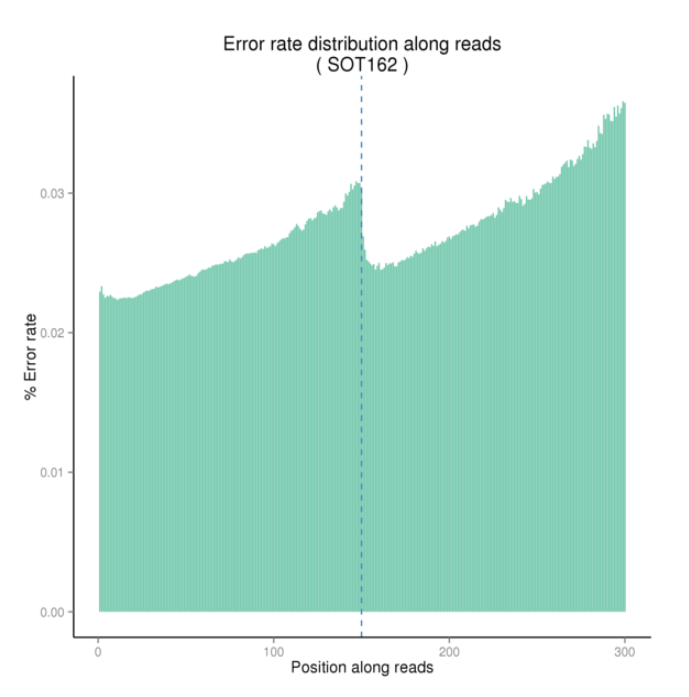


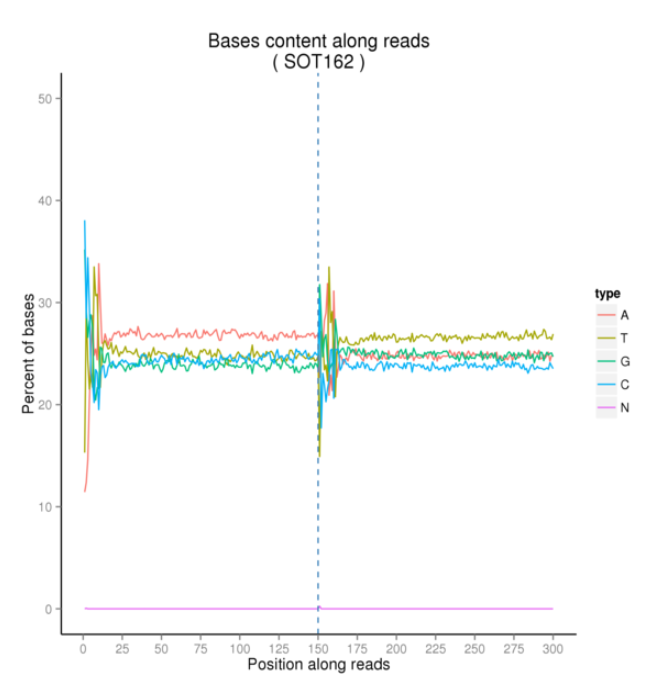

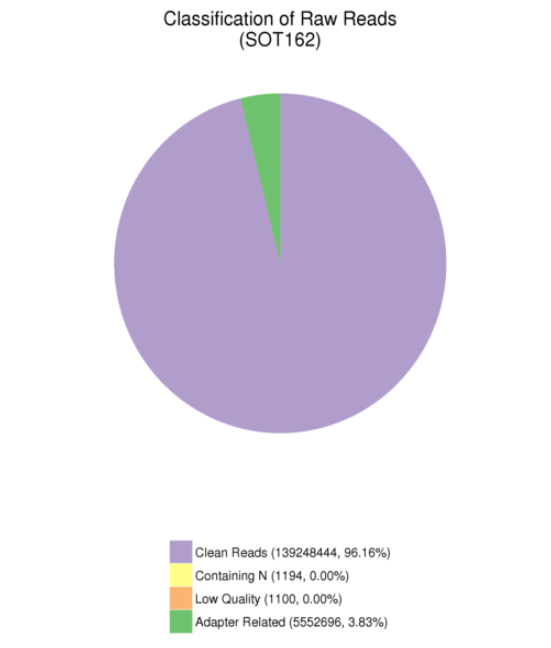


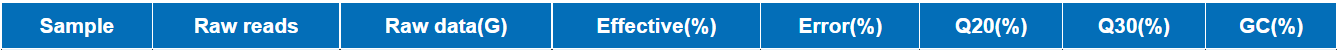

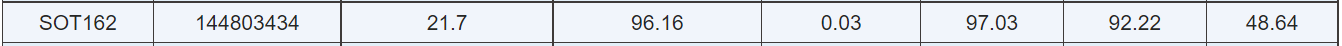


**Supporting Data D2. Primer sequences for their respective VUSs.**

| **VUS** | **Primer Sequence for short amplicon** |
| --- | --- |
| COL5A2 c.961-10T>G | GTCTTCCAGGTCTGAAGGGT |
|  | CATACCATGTGCACCTCGT |
|  |  |
| TTN c.49346-1G>A | CTGCTGTGGTGGAAGTGAAC |
|  | TTCAGCCAACACACGGAATC |
|  |  |
| TTN c.63793G>A | GGAAACGGTGTAATGCTGCA |
|  | GGATCTGATGCAAGCACTGG |
|  |  |
| SYN1 c.838-2 A>G | TCAGCAGTACAACGTACCCC |
|  | AGTTCCCTGACACTGACGT |
|  |  |
| TCF4 c.550-3C>G | GGAGGCCTCTTCACAGTAGT |
|  | CCCAACATTCCTGCATAGCC |
|  |  |
| TERT c.3295+5G>T | CCCTCTGCTACTCCATCCTG |
|  | CTCACTCAGGCCTCAGACTC |
|  |  |
| TERT c.3157+3A>G | TGAACAGCCTCCAGACGG |
|  | CAGGAGTGGCACGTAGGT |
|  |  |
| ABCA4 c.5461-10T>C | TTCATCGGCATCAACAGCAG |
|  | CCATGGCAAACAGGTTCTTCC |
|  |  |
| PRPH c.421G>T | TAGCTCTGCGAACGGTGACT |
|  | CGTGCAGCTTCTTGAGGAAC |
|  |  |
| WT1c.871A>T | CAGTTCCCCAACCACTCATT |
|  | CAGGTCATGCATTCAAGCTG |
|  |  |
| NTRK2 c.287+3G>C | AAATGCAGTGCCTCTCGGAT |
|  | CTCGTCAGTTTGTTTCGGGT |
|  |  |
| DSP c.5510A>G | TGAGGCTGGAGTACGATGAC |
|  | GCTTGGAGTCTTTCGATCTCAC |
|  |  |
| COL1A1 c.2644C>T | CTGGAGCCAAAGGTGCTC |
|  | CACCAGGGGATCCTTTCTC |

| **VUS** | **Primer Sequence for long amplicon** |
| --- | --- |
| COL5A2 c.961-10T>G | GGTGAAGTGGGATTTGCAGG |
|  | GGACCTGCTTCTCCCTTCAT |
|  |  |
| SYN1 c.838-2 A>G | TTCGGAATGGGGTGAAGGTC |
|  | GCCTGAGCCATCTTGTTGAC |
|  |  |
| TERT c.3157+3A>G | GGCACGGCTTTTGTTCAGAT |
|  | GGTCTTGAAGTCTGAGGGCA |
|  |  |
| ABCA4 c.5461-10T>C | CGGGTGAACAAATCCAAGCA |
|  | GAACTCCGACACACAGCCT |
|  |  |
| NTRK2 c.287+3G>C | GAATTGGGTTGGAGCAGGAG |
|  | GGACTGGATTTAGCCTCTTGG |
|  |  |
| COL1A1 c.2644C>T | TGAAAGTGGTCCCAGCGG |
|  | ACCTTGTTTGCCAGGTTCAC |
